# In-house reverse transcriptase polymerase chain reaction for detection of SARS-CoV-2 with increased sensitivity

**DOI:** 10.1038/s41598-021-97502-1

**Published:** 2021-09-09

**Authors:** Manash Jyoti Kalita, Kalpajit Dutta, Gautam Hazarika, Ridip Dutta, Simanta Kalita, Partha Pratim Das, Manash P. Sarma, Sofia Banu, Md. Ghaznavi Idris, Anjan Jyoti Talukdar, Sangitanjan Dutta, Ajanta Sharma, Subhash Medhi

**Affiliations:** 1 Laboratory of Molecular Virology and Oncology, Department of Bioengineering and Technology, Gauhati University, Guwahati, Assam 781014 India; 2 Department of Medicine, GMCH, Guwahati, Assam 781032 India; 3 Department of Microbiology, GMCH, Guwahati, Assam 781032 India; 4 Department of Biotechnology, Assam Down Town University, Guwahati, Assam 781068 India

**Keywords:** SARS-CoV-2, Epidemiology

## Abstract

As the COVID-19 infection continues to ravage the world, the advent of an efficient as well as the economization of the existing RT-PCR based detection assay essentially can become a blessing in these testing times and significantly help in the management of the pandemic. This study demonstrated an innovative and rapid corroboration of COVID-19 test based on innovative multiplex PCR. An assessment of optimal PCR conditions to simultaneously amplify the SARS-CoV-2 genes E, S and RdRp has been made by fast-conventional and HRM coupled multiplex real-time PCR using the same sets of primers. All variables of practical value were studied by amplifying known target-sequences from ten-fold dilutions of archived positive samples of COVID-19 disease. The multiplexing with newly designed E, S and RdRp primers have shown an efficient amplification of the target region of SARS-CoV-2. A distinct amplification was observed in 37 min using thermal cycler while it took 96 min in HRM coupled real time detection using SYBR green over a wide range of template concentrations. Our findings revealed decent concordance with other commercially available detection kits. This fast HRM coupled multiplex real-time PCR with SYBR green approach offers rapid and sensitive detection of SARS-CoV-2 in a cost-effective manner apart from the added advantage of primer compatibility for use in conventional multiplex PCR. The highly reproducible novel approach can propel extended applicability for developing sustainable commercial product besides providing relief to a resource limited setting.

## Introduction

The sudden outbreak of an acute respiratory syndrome of an unknown etiology among the population of Wuhan city in China in the month of December, 2019, appeared to be something novel in nature. In the beginning, the outbreak was presumed to have originated from a seafood market located in the vicinity of Wuhan city. Eventually, it was discovered that the causal agent of the infection was a Beta-coronavirus related to previously known members of the family of SARS and MERS. Initial reports indicated that SARS-CoV-2 was more distant from the previously known SARS-CoV and MERS-CoV than the two bat-derived SARS-like coronaviruses bat-SL-CoVZC45 (87.9% sequence identity) and bat-SL-CoVZXC21 (87.2% sequence identity). The virus belongs to the family *Coronaviridae* and order *Nidovirales* and has immense ability to mutate and undergo recombination^1–5^. SARS-CoV-2 belongs to the genus β-coronavirus which is comprised of crown-like, enveloped, positive-sense single-stranded RNA viruses. The genome sequence length of SARS-CoV-2 is about 30 kb, with a 5′-cap structure and 3′-poly-(A) tail enveloped by a complex of structural proteins to form a crown-like enveloped virus^1,6,7^. The novel 2019-nCoV caused an outbreak with a lower respiratory tract disease called novel coronavirus pneumonia (NCP), and lead to a large-scale epidemic in a short time that immediately received worldwide attention. Subsequently, 2019-nCoV was renamed as SARS-CoV-2 by the International Committee on Taxonomy of Viruses and Disease and the disease as COVID-19^8^.

Polymerase Chain Reaction (PCR) is a gold standard technique which offers significant advantages in viral detection and quantification with high reproducibility and sensitivity^9^. Initially, for the detection of COVID-19, a reliable qRT-PCR assay was developed by the Centers for Disease Control and Prevention (CDC), USA. With time, various laboratories across the world developed different primer/probe pairs for specific detection of SARS-CoV-2 with varying sensitivity and accuracy. However, in at least three independent studies it was reported that the primer pairs suggested for N region by US CDC, namely 2019-nCoV_N2 showed significant background cross reactivity^10^, along with non-specific amplification. Another primer 2019-nCoV_N3 has been reported to give false negative result^11^ in a study that includes SARS-CoV-2 positive patients besides showing false positive result even in the absence of template^10^. Similarly the most preferable primer RdRp-P2, used in more than 30 European laboratories has been reported to have less sensitivity^12^.

With the surge in disease burden, the demand for rapid screening for SARS-CoV-2 has risen significantly. Undeniably, the present circumstances have driven the need for more specific and sensitive primers for virus detection without false-positive results. We performed literature review of articles via selecting keywords ‘RT-PCR detection of COVID-19’, ‘primer specificity of SARS-CoV-2 detection assay’, ‘sensitivity and specificity of COVID-19 detection test’ with probable sensitivity limit or limit of detection (LoD) that are reported based on the analysis. Upon reviewing and analysing the primer pairs that are initially prescribed by different public health agencies, we observed a noticeable difference in detection sensitivity at lower template concentrations besides producing the false-positive results. Such limitations simultaneously drive the need for the availability of more specific primer/probes that can accurately detect COVID-19 cases in robust conditions. Moreover, the establishment of non-hazardous infrastructure encompassing skilled manpower, availability of reagent subsequently increases the per sample cost that appears to be an additional burden for any country with resource constraints.

In the current scenario, rapid and accurate detection of SARS-CoV-2 could significantly influence the management of the pandemic. World Health Organization (WHO) guideline require, at least two different targets on the COVID-19 virus genome for the detection of SARS-CoV-2 through PCR amplification. This study has therefore focused on developing a Fast Multiplex Polymerase Chain Reaction targeting E, S and RdRp gene of SARS-CoV-2, detectable through Agarose gel-based visualization besides HRM coupled multiplex Real-Time PCR based validation using the same primer set. The optimized PCR condition applied for both the traditional method and HRM coupled multiplex Real Time PCR along with the adoption of the sample pooling strategy in the present study established a more sensitive assay protocol with reduced cost.

## Materials and method

### Sample collection

Archived positive samples of a varied range of C_t_ values ranging from low to high C_t_ value (Tested using Standard Operating Procedure from ICMR-NIV Pune which uses Invitrogen SuperScript III Platinum One-Step Quantitative Kit) were collected from State Level Viral Research and Diagnostics Laboratory (VRDL), Gauhati Medical College and Hospital were used as in the study. A total of 100 known positive and 33 known negative samples were included in the study.

### Viral RNA extraction

Viral RNA extraction was carried out using AuPreP Viral RNA Extraction Miniprep System (Life technologies, Cat no: RNV-52-906LT) following the manufacturer's protocol. The extracted RNA was then quantified using Nano Drop Spectrophotometer (NanoVue plus, Make: Invitrogen) and the values recorded in ng/µl. The extracted RNA was converted into cDNA immediately.

### Reverse transcription

Tetro cDNA Synthesis Kit (Make: Meridian Bioscience, Cat. No: BIO-65043) was used for cDNA preparation using the manufacturer’s protocol. 5 µg of the extracted RNA was used for cDNA preparation in a final volume of 20 µl reaction. Each reaction was incubated at 25 °C for 10 min followed by 45 °C for 30 min and finally terminated by incubating at 85 °C for 5 min.

### Primer design

The full sequences of SARS-CoV-2 were retrieved from the NCBI Reference Sequence Database. Alignment of the sequences was done in BioEdit software. Primer3 tool (https://bioinfo.ut.ee/primer3-0.4.0/) was used to design the three primer sets targeting the SARS-CoV-2 specific E gene, S gene and RdRp gene. The selected primer pairs were analyzed using NCBI primer blast for the specificity of the primer set. Blast report showed that all the three primers have specifically amplified the target region of SARS-CoV-2 only. The primers pairs were further analyzed for secondary structure, the amplicons as well as probable self and heterodimer formation tendencies using Idtdna.com (https://sg.idtdna.com/pages/tools/oligoanalyzer). The primer sets were synthesized and delivered by Reprocell Brand: Bioserve (Hyderabad, Telangana. India).

### Primer testing and development of fast PCR assay

The accuracy and optimization of each primer set was verified through PCR amplification. Gradient PCR of the test was performed with an annealing temperature profile ranging from 62 to 68 °C based on the melting temperature (T_m_) of each of the SARS-CoV-2 specific target primer set. The template concentration was kept up to a maximum of 10 ng of cDNA in a reaction volume of 20 μl along with forward and reverse primer sets at a final concentration of 0.2–0.3 μM each using Emerald Amp GT PCR Master Mix (2x) from Takara Bio Inc. (Cat No. RR310A). The cycling condition includes an initial denaturation step at 95 °C for 5 min. Amplification was carried out for 35 cycles with denaturation at 95 °C for 20 s, followed by a temperature gradient of 62–68 °C for 30 s to obtain the optimum annealing temperatures, and extension at 72 °C for 20 s. A final extension was carried out at 72 °C for 7 min.

Following primer optimization, the cycling condition was modified by reducing the denaturation time to 5 s and eliminating the extension step at 72 °C for 20 s. As such the Fast PCR assay will involve an initial denaturation at 95 °C for 5 min. Amplification will be done by denaturation at 95 °C for 5 s, followed by annealing at 66 °C for 15 s and extension at 72 °C for 5 min.

### Fast multiplex PCR amplification of E, S and RdRp gene

To minimize the amount of sample and reagent usage, preparation time, cost and labor, we developed an alternative protocol by adopting a multiplex PCR protocol for detection of SARS-CoV-2 in which all the primer sets were mixed into one reaction which reduced the total number of reactions to 1 (one) per sample instead of 3 (three) reactions. When mixed, the final concentration of the primer pairs was reduced proportionally to decrease primer- dimer formation. The designed primer pairs targeting the E, S and RdRP genes in the SARS-CoV-2 genome, produced amplicons of different sizes (i.e., 101 bp, 103 bp, and 160 bp, respectively) for easy separation and visualization. The Applied Biosystems Veriti 96-Well Thermal Cycler instrument was used for multiplex PCR. EmeraldAmp GT PCR Master Mix (Cat No: RR310A) was used and each 20 µl reaction mixture contained 10 µl of PCR Master Mix 0.5 µl of each primer and 1 µl of synthesized cDNA. The final concentrations of primers were 0.2 µM (E,S and RdRp gene primers). The thermal cycler was set as per the temperature profile for the Fast PCR assay described earlier.

### Sensitivity and specificity of multiplex PCR

The sensitivity of the primer pairs was tested using multiplex PCR with optimized primer pairs for E, S and RdRp gene on archived known SARS-CoV-2 positive and negative samples. Variable C_t_ values of the samples ranging from 19 to 35 makes it possible to analyze the sensitivity of the primer pairs. Apart from this the template was diluted 10 times serially and tested for sensitivity and specificity of the primer pairs using multiplex PCR.

#### Cross reactivity analysis

To analyse the cross reactivity of the assay procedure with other respiratory viruses, the multiplex PCR was tested against archived Influenza A positive, Influenza B positive and H1N1positive samples. However, as an efficient alternative cross reactivity studies can be performed in-silico where cross reactivity can be defined as 80% or more sequence similarity between a primer and any nucleotide sequence in the target organism^13^.

### Multiplex high resolution melting (HRM) coupled real time PCR for detection of SARS-CoV-2

100 nanogram of previously synthesized cDNA from archived known SARS-CoV-2 positive samples was used with TB Green Premix Ex Taq II (Takara, RR820B) using Rotorgene Q (Qiagen) 5plex Real Time PCR machine following cycling condition of an initial hold at 95 °C for 5 min; followed by a 45 repeat cycle of hold at 95 °C for 20 s, annealing at 66 °C for 30 s, final extension at 72 °C for 20 s. High Resolution Melt profile was set for a range of temperature from 55 to 95 °C with 0.1° gradual increments. Standardized primer pairs were used at a final concentration of 0.2 µm for simultaneous detection of E, S and RdRP genes in the SARS-CoV-2 genome.

### Sequencing and phylogenetic analysis

PCR amplicons of E, S and RdRp gene were sequenced through Sanger sequencing (Bioserve sequencing service from Reprocell USA, inc, Hyderabad, Telangana. India). The obtained sequence was aligned separately for the three different gene targets against the SARS-CoV-2 reference sequence (NC_045512) and other related coronavirus sequences obtained from the NCBI database to analyse the percent identity.

### Compliance statement

All the experimental procedures were performed in accordance with the relevant guidelines and regulations provided by Indian Council of Medical Research (ICMR) which is again in accordance with WHO laboratory safety manual related to the novel coronavirus (2019-nCoV) (https://www.who.int/docs/default-source/coronaviruse/laboratory-biosafety-novel-coronavirus-version-1-1.pdf?sfvrsn=912a9847_2). All the samples were appropriately processed and maintained in a well-ventilated Biosafety cabinet by personnel with demonstrated capability. All bio hazardous waste generated were disinfected using Sodium Hypochlorite (0.1%) for general disinfection and 1% hypochlorite solution was used for any kind of spillage during sample processing. The entire work was done in the dedicated Biosafety level-3 (BSL-3) facility of State Level Viral Research and Diagnostics Laboratory (VRDL) Gauhati Medical College and Hospital. This study also has the ethical approval of the Institutional Ethical Committee of Gauhati Medical College & Hospital, Guwahati, Assam-781032 vide ethical approval letter no: MC/190/2007/Pt II/Oct. 2020/14.

### Ethical clearance

The study has been ethically approved by the institutional ethical committee of Gauhati Medical College & Hospital, Guwahati, Assam-781032 vide ethical approval letter no: MC/190/2007/Pt II/Oct. 2020/ 14.

## Results

### Primer optimization

**Figure 1:**
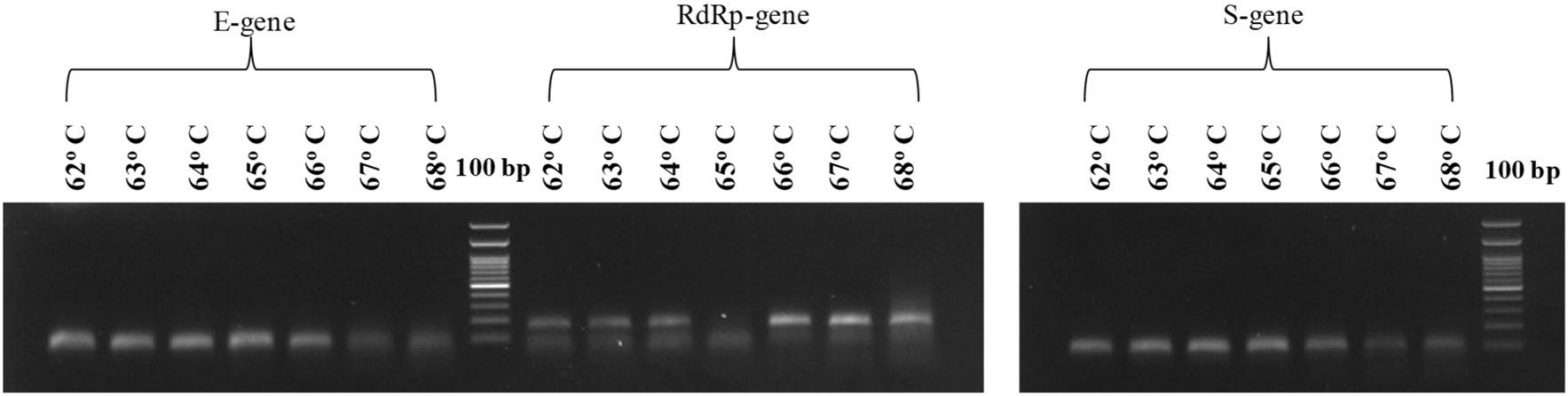
Amplification of E, RdRp and S genes at different temperature in gradient PCR. (Lane 1–7 E-gene, Lane 8 100 bp ladder, Lane 9–15 RdRp-gene, Lane 16–22 S-gene, Lane 23 100 bp ladder.

In-silico validation of primer pairs and amplicon sequences showed no secondary structure and the possibility of self or heterodimer formation was not observed. In silico PCR tool nullified the possibility of nonspecific reactions within the same genome as well as genomes from other related species. The optimum annealing temperature of primer pairs was found to be 66 °C. The optimum primer concentration for multiplex PCR was found to be 0.2 μM final concentration of each primer. With this primer concentration, the resulting Agarose gel image showed no primer dimer formation (Fig. 1).

### Fast PCR protocol for SARS-CoV-2 detection

**Figure 2:**
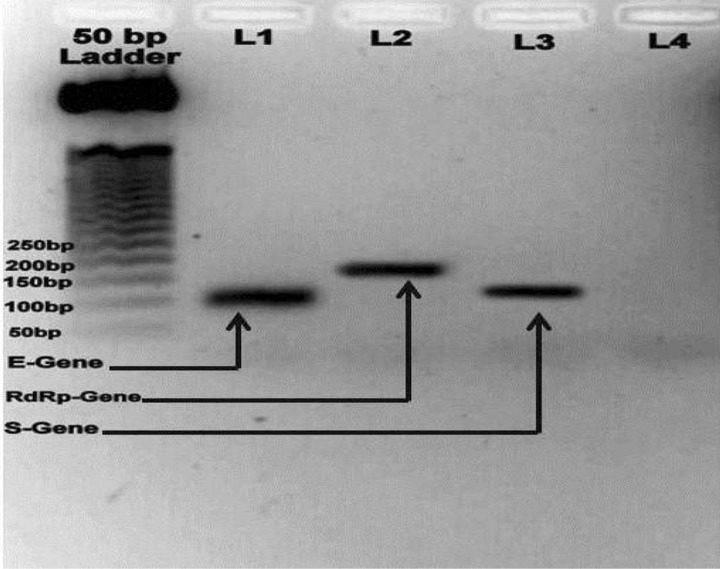
Fast PCR protocol for SARS-CoV-2 detection. Gel electrophoresis results from PCR (35 cycles) showing amplification of RdRp-Gene (Lane-2, L2), S-Gene (Lane-3, L3) and E-gene (Lane-1, L1), and Negative Control (Lane-4, L4) respectively. Ladder used 50 bp DNA ladder (Promega, Cat. No: G4521).

Following primer optimization, all three primers were tested separately using a single known positive sample with our optimized PCR cycling condition to observe the amplification efficiency of the protocol. Agarose gel electrophoresis showed distinct band of size 101 bp, 103 bp, and 160 bp for E gene, S gene and RdRp gene respectively without any primer dimer formation (Fig. 2).

### Development of a fast multiplex PCR protocol for SARS-CoV-2 detection

**Figure 3:**
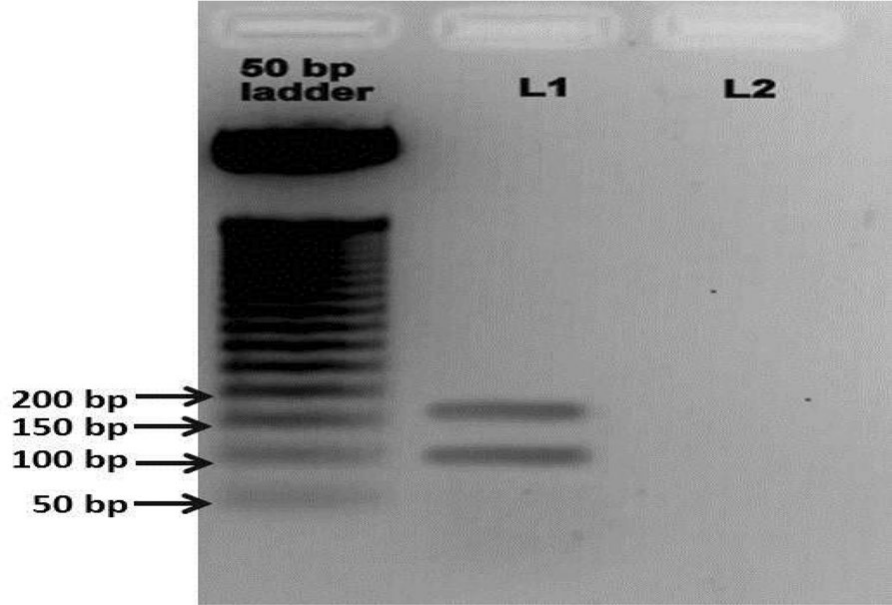
Fast Multiplex PCR protocol for SARS-CoV-2 detection: gel electrophoresis results obtained from multiplex PCR in the SARS-CoV-2, showing amplification of two genes (RdRp Gene and E gene) Since E gene and S gene product are overlapping. Ladder used 50 bp DNA ladder (Promega, Cat. No: G4521).

We developed a Fast PCR protocol for SARS-CoV-2 detection and easy implementation in any biological laboratory in the world. Archived positive samples from State Level Viral Research and Diagnostics Laboratory (VRDL) Gauhati Medical College and Hospital was used for cDNA preparations and used as a template for multiplex PCR optimization. The PCR was performed for 35 cycles following the cycling conditions described earlier. The gel electrophoresis showed two sharp bands of variable size i.e. 160 bp for the targeted RdRp gene and another of two overlapping bands of 101 bp and 103 bp for the E gene and S gene of SARS-CoV-2 respectively (Fig. 3).

### HRM coupled multiplex RT-PCR based detection of SARS-CoV-2

**Figure 4:**
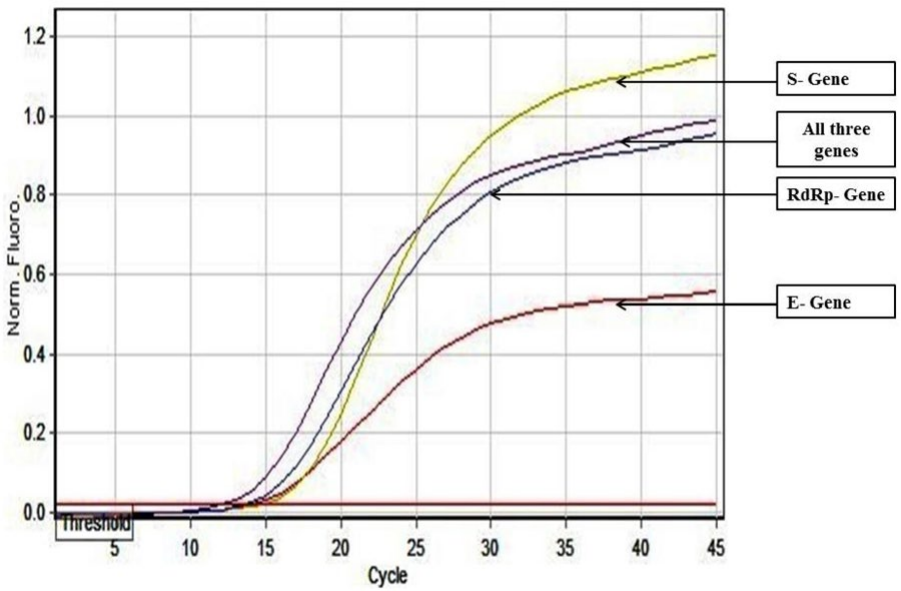
Amplification graph of E, S and RdRp gene of SARS-CoV-2 using SYBR green master mix (TB Green Premix Ex Taq II, Takara, Cat. No: RR820B). All the three primers were tested individually as well as in multiplexing reaction in single tube. Amplification plot showed efficient amplification of the three gene individually and also upon multiplexing of the three primers.

**Figure 5:**
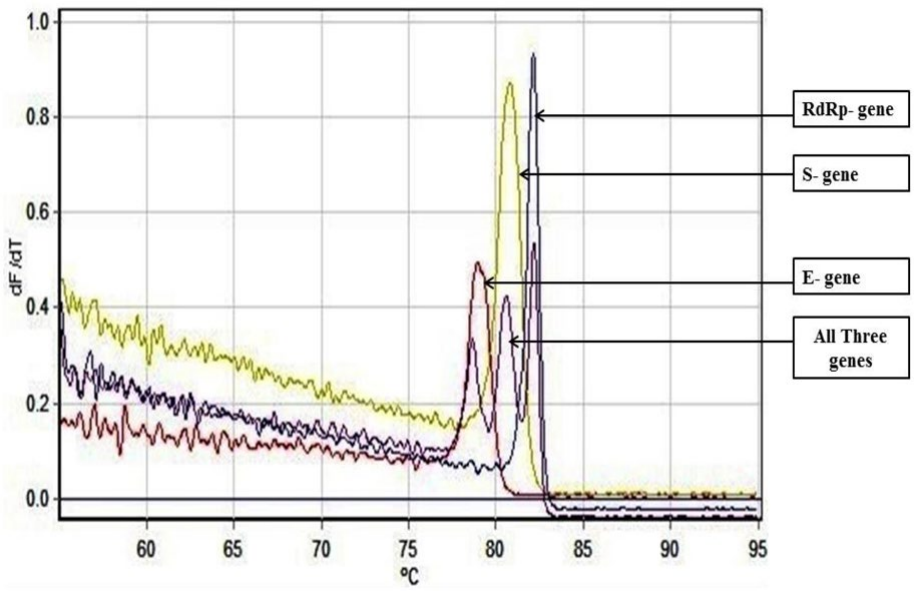
High Resolution Melting Curve plot for the amplicons of E, S and RdRp gene. All the three amplicons showed distinct well separated melting peak both in multiplexing reaction and in individual reaction condition.

**Figure 6:**
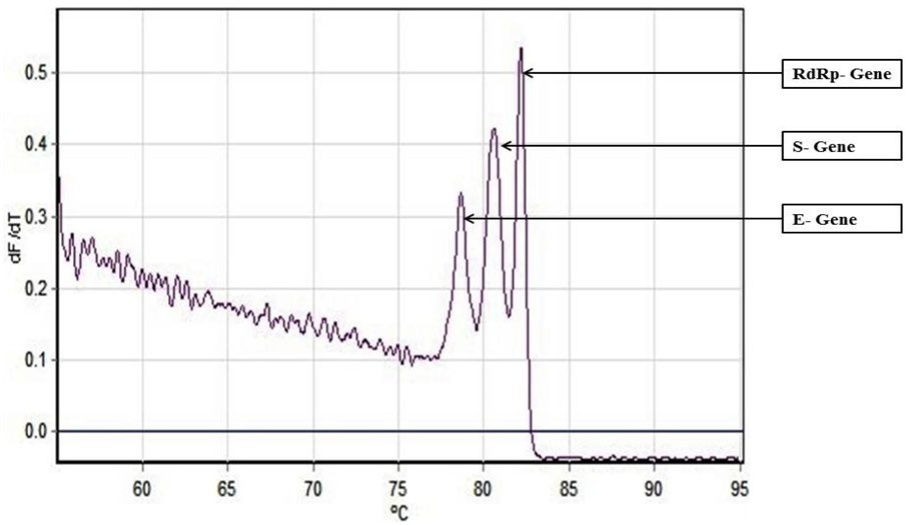
Well separated melt curve peak for E, S and RdRp gene upon multiplex RT-PCR.

**Figure 7:**
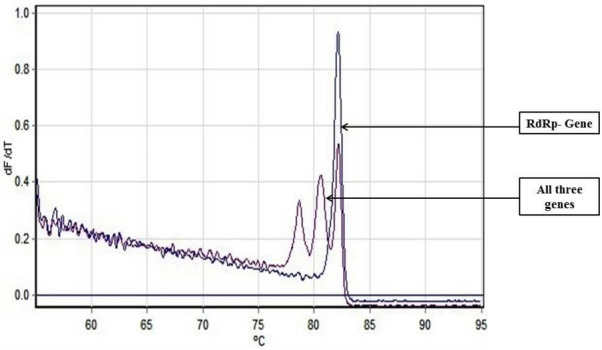
Individual melt curve peak for RdRp gene obtained upon single-plex RT-PCR reaction using primer pairs for RdRp gene.

**Figure 8:**
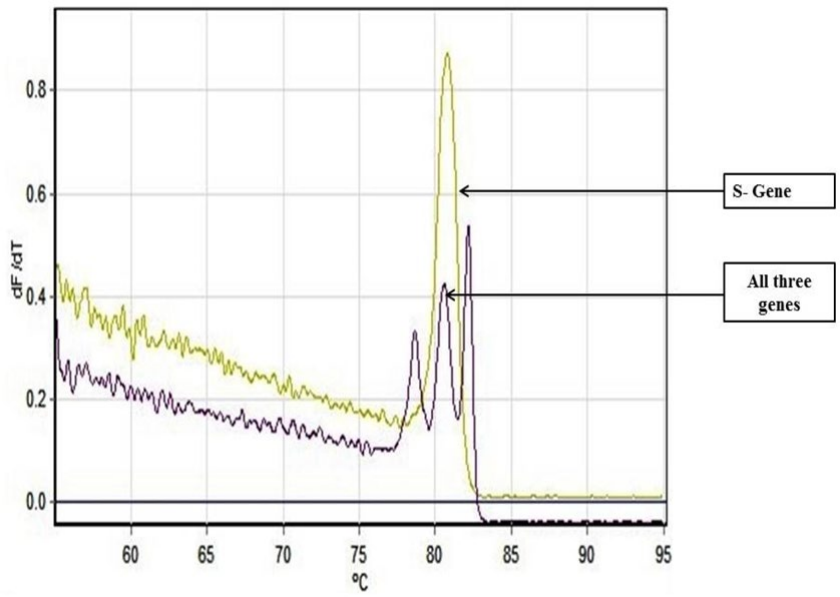
Individual melt curve peak for S gene obtained upon single-plex RT-PCR reaction using primer pairs for S gene.

**Figure 9:**
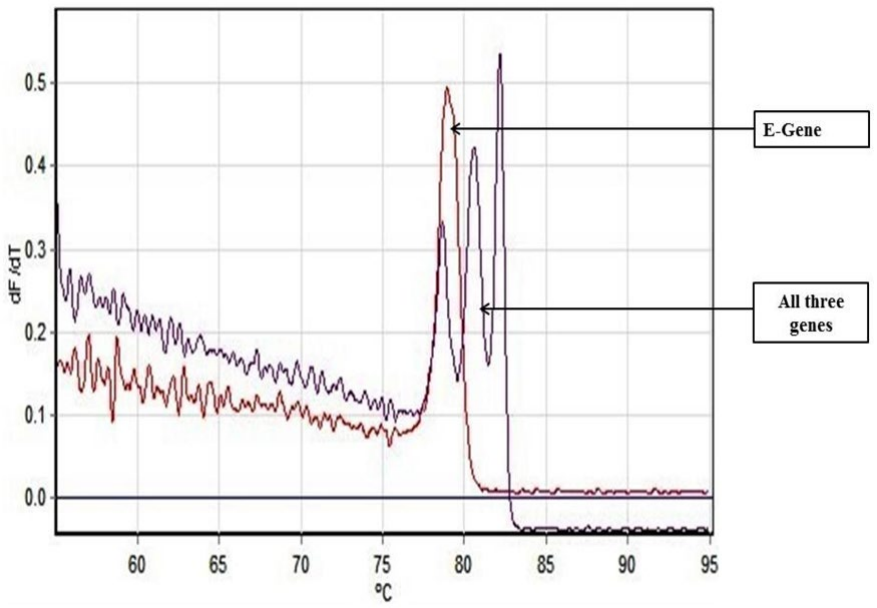
Individual melt curve peak for E gene obtained upon single-plex RT-PCR reaction using primer pairs for E gene.

As an alternative to our Fast Multiplex PCR protocol for SARS-CoV-2 detection, we further developed a multiplex RT PCR assay with implementation of High-Resolution Melting profile in addition to the thermal cycling profile for obtaining an enhanced separation of melt curve. A very similar result was obtained for HRM coupled multiplex RT PCR assay. The amplification plot showed one combined reaction curve with significantly lower C_t_ value for all the three primers put together apart from the amplification curve for the individual primer put in separate reaction tube (Fig. 4). The accuracy of the multiplex reaction in real time was confirmed further by analyzing the high-resolution melt curve with three distinct peaks (Fig. 5) which were further compared with the melt curve peak obtained for the three primers when used separately in distinct tubes (Figs. 6, 7, 8, 9). Based on these observations we can conclude that the three distinct melt curve peaks were of E, S and RdRp genes. Thus, this result demonstrates that the newly developed HRM coupled multiplex RT PCR protocol can be used for the fast and accurate detection of SARS-CoV-2 without the need of a costly probe based approach of detection.

### Sensitivity and specificity

The multiplex RT PCR assay successfully amplified all the three targets with distinct melt curve (E, S and RdRp gene) without any false results upon testing with known positive archived samples of SARS-CoV-2 having C_t_ values ranging from 19 to 35 obtained with other commercial kits. A C_t_ value < 30 was obtained for N = 100 (100%) archived SARS-CoV-2 positive sample inclusive of different age and gender. This is comparable with the recommended maximum C_t_ value for positive interpretation of COVID-19 using commercially available approved RT-PCR based detection kit (C_t_ ≤ 35), and as such the number of true positive (TP) sample was obtained as 100 (100%) and the number of true negative (TN) sample was 33 (100%) without any false result. Thus the sensitivity and specificity was obtained as *Sensitivity* = *TP/(TP* + *FN)* = *100/(100* + *0)* = ***1*** and *Specificity* = *TN/(TN* + *FP)* = *33/(33* + *0)* = ***1.*** This is suggestive of the enhanced sensitivity of the HRM coupled multiplex RT PCR assay.

#### Cross reactivity of the assay

There were no false positive results from any of the primer pairs for the three target genes when the cross reactivity of the assay procedure was analysed with archived Influenza A, Influenza B and H1N1 positive samples. In-silico cross reactivity analysis for all the three primer pairs further showed 100% SARS-CoV-2 specific hit^14–16^.

#### Limit of detection (LoD)

**Figure 10:**
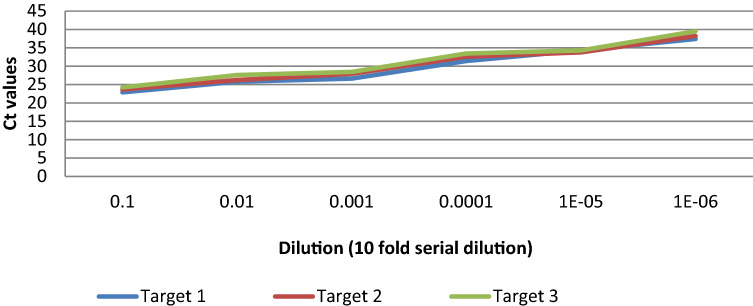
Graph showing average C_t_ value obtained from five replicates for a series of 7, tenfold serial dilution of an initially tested SARS-CoV-2 positive sample. All replicates were detected up to 10^–5^ dilution. No replicates were detected at 10^–7^ dilution.

LoD was determined by using a tenfold serial dilution of the clinical sample SC005G (4.71 × 10^5^ RNA copies/ml), which was previously reported to have a low C_t_ value (*C*_*t*_*- ORF1b* = *19, C*_*t*_- *N gene* = *21; Meril COVID-19 detection kit).* All the dilutions were replicated 5 times and the average C_t_ value obtained for individual target region for each dilution has been shown in Table 1 All the three targets were detected in 100% of the replicates for dilution ranging from 10^−1^ (4.71 × 10^4^ RNA copies/ml) to 10^–5^ (4.71 RNA copies/ml) with a corresponding average C_t_ value of 34.28 for RdRp gene, C_t_ value of 33.77 for E gene, and C_t_ value of 34.30 for S gene (Fig. 10). At 10^–6^ dilution, Target 1 and Target 2 were detected in 40% of the replicates with average C_t_ value of 37.42 for Target 1 (RdRp gene) and 38.21 for Target 2 (E-gene). Target 3 (S-gene) was detected in 60% of replicates with average C_t_ value of 39.53 as shown in Table 1. However, none of the target was detected in any of the replicates for 10^–7^ dilution (Table 1).

**Table 1:** Average C_t_ values obtained for three target regions from a series of 7 tenfold serial dilution of a clinical specimen previously detected SARS-CoV-2 positive (SC005G) using Meril covid-19 detection kit.

Target 1 (RdRp)				Target 2 (E-gene)			Target 3 (S-gene)		
Dilutions	Replicates detected	Average CT-values	SD	Replicates detected	Average CT-values	SD	Replicates detected	Average CT-values	SD
10^–1^	5/5(100%)	22.87	0.553968	5/5(100%)	23.67	0.915341	5/5(100%)	24.25	0.604276
10^–2^	5/5(100%)	25.77	0.663928	5/5(100%)	26.22	0.663099	5/5(100%)	27.62	0.934532
10^–3^	5/5(100%)	26.62	0.703314	5/5(100%)	27.92	0.557692	5/5(100%)	28.43	0.79117
10^–4^	5/5(100%)	31.42	1.406722	5/5(100%)	32.58	0.978673	5/5(100%)	33.45	0.41142
10^–5^	5/5(100%)	34.28	0.407799	5/5(100%)	33.77	0.721249	5/5(100%)	34.30	0.44238
10^–6^	2/5(40%)	37.42	1.286934	2/5(40%)	38.21	0.59397	3/5(60%)	39.53	0.600083
10^–7^	0/5(0%)	–	–	0/5(0%)	–	–	0/5(0%)	–	–

#### Experimental C_t_ values obtained for target 1, 2 and 3 among the positive specimens

All the 100-specimen showed reportable C_t_ values for all the three targets. Mean C_t_ value for Target 2 was found to be lowest among the tested SARS-CoV-2 positive samples followed by Target 1 and Target 3. However considerable degree of similarity and closeness of C_t_ values among the three targets were observed. When stratified by age, within age group 80–89 years, the C_t_ value for, Target 1 was 4.98 C_t_ lower than the overall mean C_t_ for Target 1, for Target 2 it was 5.06 C_t_ lower and for Target 3 it was found to be 4.37 C_t_, lower than the overall C_t_ value (Table 2) (Supplement Figure 1), which is a representative of high viral load in this age group.

**Table 2:** Mean C_t_ values obtained for the entire three targets among the positive specimens, stratified by age.

Group	No of specimen	Target 1(RdRp gene)	Target 2 (E gene)	Target 3 (S gene)
		Mean C_t_	Mean C_t_	Mean C_t_
All specimen	100	24.53	23.96	25.37
Age < 10 y	2(2%)	22.56	24.05	25.76
Age 10–19 y	2(2%)	25.7	24	25.7
Age 20–29 y	4(4%)	26.50	26	25.7
Age 30–39 y	7(7%)	23.3	23	23.8
Age 40–49 y	10(10%)	26.3	25.7	27.4
Age 50–59 y	31(31%)	24.79	24.2	25.5
Age 60–69 y	29(29%)	24.74	24.4	25.9
Age 70–79 y	11(11%)	23.1	21.9	24.01
Age 80–89 y	4(4%)	19.55	18.9	21
Age > 89 y	0	0	0	0

Age group 80–89 showed considerably lower C_t_ value for the entire three targets in comparison to mean C_t_ which is representative of high viral load.

#### Comparison of sensitivity and specificity of our In house RT-PCR assay

- Comparison of C_t_ value

**Figure 11:**
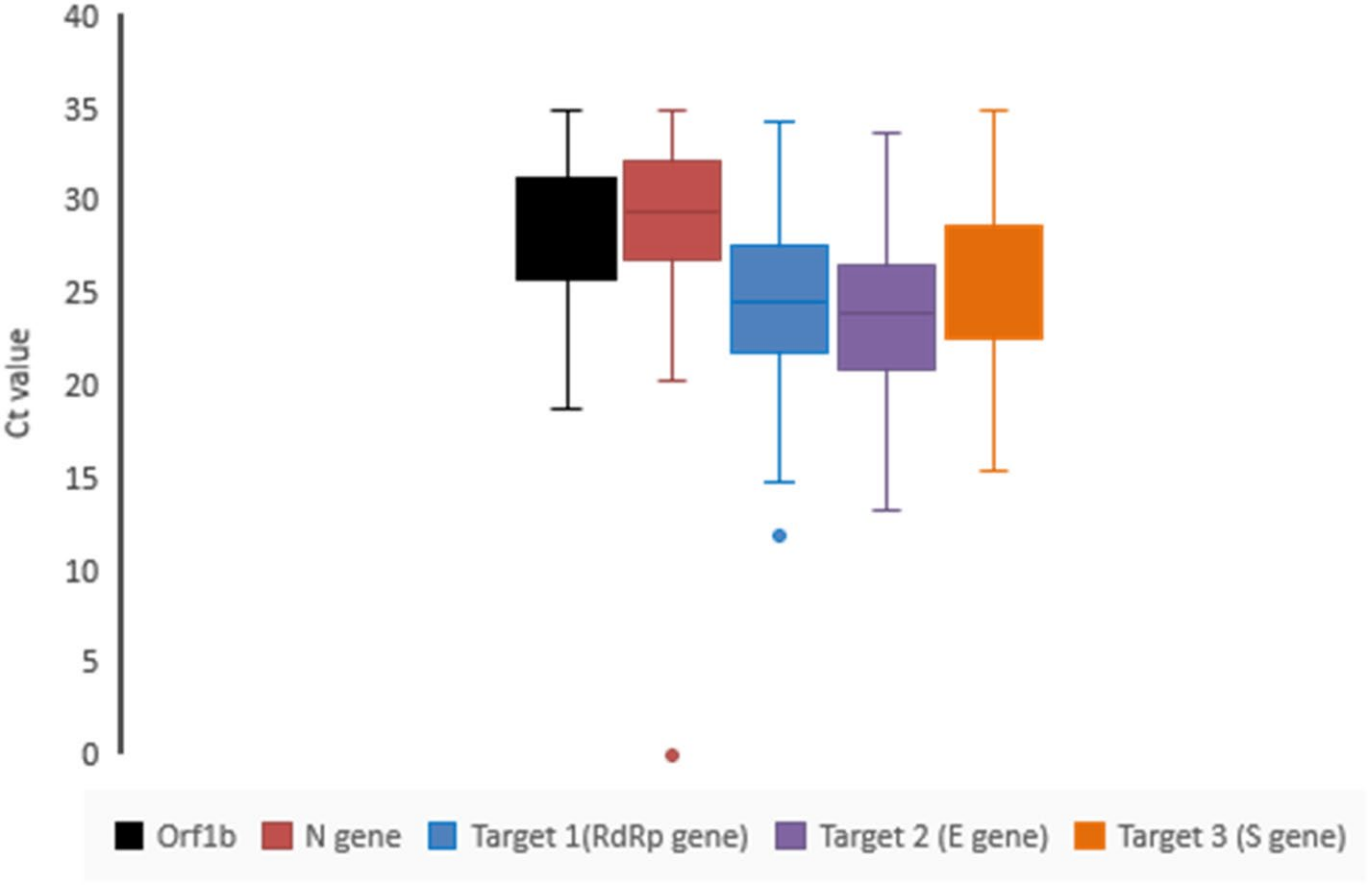
Box plot diagrams representing the C_t_ values obtained for N = 100 samples previously tested SARS-CoV-2 positive with Meril Covid-19 detection kit. Dark bar: median; box: 25–75% interquartile; whiskers and dots: range in the box plot. Significant difference in C_t_ values was observed upon testing with In house SARS-CoV-2 detection assay (Target 1, Target 2 and Target 3) when compared with that of Meril Covid-19 detection kit (Orf1b and N gene). Enhanced sensitivity of the in-house RT-PCR assay is evident from the plot showing comparable reduction in median C_t_ value.

The sensitivity and specificity of the primer pairs were further analysed by comparing the C_t_ value obtained through our optimized detection protocol with the C_t_ value obtained for the same set of samples (N = 100) using commercially available detection kits used in diagnostic setup (Meril COVID-19 One-Step RT-PCR Kit; Cat no: NCVPCR-02). The average C_t_ value was found to be ≤ 30 for all the three target (Supplement  Table 1) which is comparable with the Meril COVID-19 RT-PCR kit (C_t_ value ≤ 35, positive) with significantly reduced median C_t_ value obtained with our In house HRM coupled RT-PCR assay (Fig. 11).

(b) Comparison of limit of detection (LoD)

**Figure 12:**
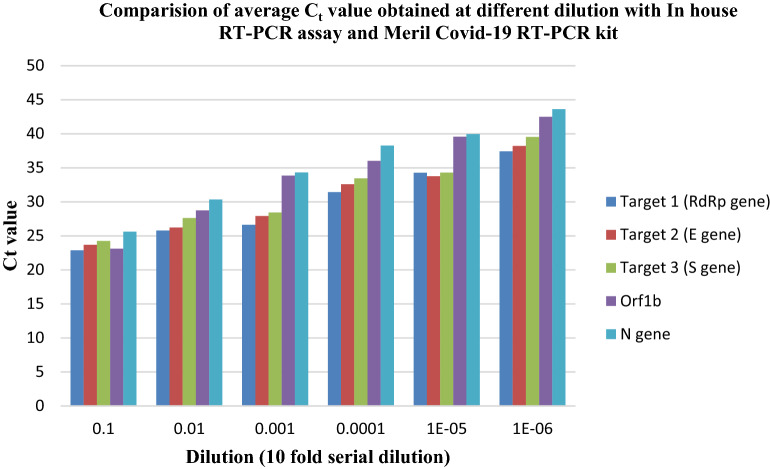
Comparision of average C_t_ value obtained at different dilution (tenfold serially diluted) with In house RT-PCR assay and Meril COVID-19 RT-PCR kit. Although efficient amplification was obtained upto 10^–6^ dilution with both the assay, C_t_ value ≥ 35.

Upon analysing the Limit of Detection (LoD) for Meril COVID-19 RT-PCR kit using the same serially diluted sample, efficient amplification of the prescribed target Orf 1b and N gene was obtained for the dilution range from 10^–1^ to 10^–3^ with C_t_ value ≤ 35 for both the target. As such the limit of detection was found to be 471 RNA copies/ml (Fig. 12). Although amplification was observed for further diluted sample (10^–4^ to 10^–6^) but the C_t_ value obtained followed a gradual increase pattern from the recommended C_t_ value for interpretation of SARS-CoV-2 positive detection (Supplement Table 2). No amplification was observed at 10^–7^ dilution. However efficient amplification up to 10^–5^ dilution (approximately 5 RNA copies per reaction, C_t_ value < 35) using our In house RT-PCR assay is suggestive of enhanced limit of detection of the assay.

#### Risk of appearing false-negative result in the assay

**Figure 13:**
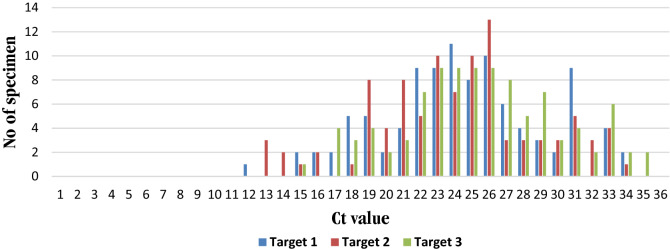
Distribution of experimentally obtained C_t_ values for the 100 positive specimen for Target 1, Target 2, and Target 3 are plotted. Only 2/100 (2%) positive sample were found with C_t_ values slightly higher than LoD or near the assay LoD and as such the sensitivity of detection may be less than 100%. Remaining 98 specimens showed efficient detection of the entire three targets.

Our tenfold serial dilution study showed the assay LoD to be 10^–5^ dilution (4.71 RNA copies) with corresponding C_t_ value of 34.28 for Target 1, 33.72 for Target 2, and 34.3 for Target 3. At 10^–1^ dilution 100% of the specimens were detected for the entire three targets. As such, the risk of false negative result increases from 0% at 10^–1^ dilution (approximately 11 C_t_ lower than LoD) to 95% at LoD, which increases further with increase in C_t_ value after LoD. In the studied cohort of SARS-CoV-2 positive samples Mean C_t_ value for Target 1(24.53) and Target 2 (23.96) was 10 C_t_ below the LoD, and for Target 3, mean C_t_ (25.37) value was 9 C_t_ lower than the LoD value. Only 2/100 (2%) positive samples showed a slightly higher C_t_ value only for Target 3 (approx. 35) than the LoD, whereas for Target 1 and 2, the obtained C_t_ values were near the assayed LoD, and as such would be at risk of false negative result. However, the assay efficiently detects 98/100 (98%) samples for Target 1 and Target 2, thereby supporting the enhanced sensitivity for RdRp gene (Target 1) and E gene (Target 2) detection (Fig. 13).

### The time required for the assay

The extraction of RNA from the sample will depend upon the type of kit or protocol used. Most of the commercially available kit requires around 35–45 min for RNA extraction. In the present study, following the RNA extraction, cDNA synthesis required 45 min with the Tetro cDNA synthesis kit (Meridian Bioscience, BIO-65043) followed by PCR amplification of the target genes from SARS-CoV-2 which required 37 min. Subsequently Agarose gel-based visualization needed approximately 30 min. Thus, the Fast PCR assay protocol needed 67 min after RNA extraction and cDNA synthesis. With the HRM coupled multiplex RT-PCR based detection, a time of 96 min was needed post RNA extraction and cDNA synthesis.

## Discussion

Targeting the nucleic acid evaluation of any living microbial entities is considered the gold standard for the detection of any active infection. The advantage of PCR-based viral detection relies on high reproducibility, sensitivity and ability to quantify vial load with accuracy^9^. In the present study, we developed three sets of primers encompassing the E gene, RdRp gene and S gene of the SARS-CoV-2 genome with enhanced sensitivity and specificity. The primers sets were designed with the additional objective of extended feasibility to further amplify in both gel-based multiplex PCR assay and SYBR green based real-time detection in a single tube. The same sets of primer produced distinct amplification in both conventional as well as HRM coupled multiplex RT-PCR based detection assay. Indeed, with optimized primer concentration and low template concentration, this study seems to provide a cost-effective detection protocol with accuracy, which opens up prospective vistas to develop and commercialize a new detection kit. However, developing a fast-track detection kit from our study protocol needs further research with absolute refinement and necessary modification. Owing to its highly sensitive primer pairs, the assay procedure offers versatility in its application in variable resource settings for efficient and cost-effective detection of SARS-CoV-2. Such an innovative approach can additionally set a significant impact on the detection and management of the COVID-19 pandemic and is useful to prepare for the future onset of any acute viral infection of similar nature and magnitude.

The present study has shown promising results in the cross-reactivity assay and Limit of Detection (LoD) analysis. Non-amplification of our targeted genes in Influenza A, Influenza B and H1N1 positive samples further reduces the chance of cross-contamination and diminished the risk of getting false-positive results. Moreover, LoD analysis of the samples up to 10^–5^ dilution with 100% efficacy revealed high specificity of the primers even in low template concentration (approximately 5 RNA copies per reaction (C_t_ value < 35) provides added advantages besides exhibiting an obvious comparison with other published assay. Supportive evidence came from the study of Garg et al.^17^ which reports that Fosun COVID-19 RT-PCR kit have the lowest LoD (300 Copies/ml, C_t_ < 36) among the seven RT-PCR kit evaluated in the study. However, the efficacy of the reaction reduces considerably in 10^–6^ dilution may be due to exhausting template concentration. Further obtaining a C_t_ value ≤ 30 for all the three target in (N = 100) archived positive sample without any false result with our in-house RT-PCR assay is suggestive of its enhanced sensitivity and specificity. Significant variation in the C_t_ values was observed from the original sample C_t_ value derived from COVID-19 positive cases, which indicates the possibility of having varied specificity of the original SARS-CoV-2 detection kit (Meril COVID-19 detection kit, C_t_ ≤ 35, positive)). The penultimate evaluation of the study has been established due to reportable and reproducible C_t_ value that extensively justified our study design. Highest deviation of C_t_ was observed in the segregated age group of 80–89 years from mean C_t_ value of the three target genes by about 4–5 unit, that might be due to pipetting error or sample handling error during experiment setup.

Initially approved SARS-CoV-2 RT-PCR test kit developed by various manufacturers around the world targeted the different number of genes, embedding three and two or even single gene with or without internal control. Accordingly, a diverse range of cost, specificity and efficacy of the kits were observed. For instance, Gene Matrix (USA), Daan Gene Co. Ltd (China), Kogene Biotech (Korea), LabGenomics (South Korea), Primer Design (UK), SansureBiotechInc. (China) used either combination of N, RdRP or E, RdRP or ORF1ab, N probe/primer in the kit^18,19^. However, all the RT-PCR based kit possess limitation in terms of cost of reagent, reporter dye and synthesis of high-end specificity probe. The cost of the probe-based RT-PCR test is an obligatory limiting factor of COVID-19 management that lay a huge financial burden for any resource-limited country in the world^13^. For most of the presently available RT-PCR assay kits, the estimated cost is around $ 40–50 (Centres for Medicare and Medicaid Services, COVID-19 test pricing. 2020)^14^. Moreover, some detection assays possess constraints both in terms of reagent limitation and low specificity with initial template concentration. Apart from that, whether constant mutation of the SARS-CoV-2 genome is influencing the efficacy of probe-based detection is still not revealed entirely and that needs profound research. However, our detection assay involving the implementation of high-resolution melting analysis following multiplex PCR amplification using SYBR green master mix further reduces the cost significantly by eliminating the need for probe, and cut down the cost to around $ 13–15.

The first recommended detection kit in India was developed and supplied by the National Institute of Virology (NIV), Pune, which used the SuperScript III One-Step RT-PCR System with Platinum Taq DNA Polymerase (make Invitrogen Catalogue number: 12574026) that requires about 5–6 h for confirmation of the result. While various other commercial kits are available that target major genes of SARS-CoV-2 requires a time of around 1.0 to 3.5 h after sample preparation. In contrast, our protocol observed successful amplification in 37 min using Fast multiplex PCR and 96 min in HRM coupled real-time detection using SYBR green that reduces the time by around 2–threefold. Additionally, the sample pooling strategy can further contribute to a reduction of cost. In addition to that, sample pooling strategy can further reduce the cost per sample in an emergency^20^. However, this approach requires robust standardization as the probability of reducing efficacy due to the exhaustive PCR component seems to be a major limitation associated with the experiment strategy. With the demonstration of absolute reproducible results with our detection assay involving innovative modification of conventional SARS-CoV-2 test protocol, the HRM coupled multiplex RT-PCR assay represents an easy, fast and low-cost option for SARS-CoV-2 detection with resource limitation.

## Supplementary Information

Supplementary Information 1.

Supplementary Information 2.
