# Supplementary material for: In-house reverse transcriptase polymerase chain reaction for detection of SARS-CoV-2 with increased sensitivity
Source: Sci Rep. 2021 Sep 9;11:17878. doi: 10.1038/s41598-021-97502-1 (PMC8429455; doi:10.1038/s41598-021-97502-1)
Supplement: Supplementary file 1 — Supplementary Information 1. [file 41598_2021_97502_MOESM1_ESM.docx]

**In-house Reverse transcriptase polymerase chain reaction for detection of SARS-CoV2 with increased Sensitivity.**

Manash Jyoti Kalita^Ϯ,1^, Kalpajit Dutta^Ϯ1^, Gautam Hazarika^1^, Ridip Dutta^3^, Simanta Kalita^1,2^, ParthaPratim Das^1,2^, Manash P Sarma^4^, Sofia Banu^1^, Md. Ghaznavi Idris^1^, Anjan Jyoti Talukdar^2^, Sangitanjan Dutta^2^, Ajanta Sharma^*3^, SubhashMedhi^**1^

**^1^**Department of Bioengineering and Technology, Laboratory of Molecular Virology and Oncology, Gauhati University, Guwahati,Assam-781014

^2^Department of Medicine, GMCH, Guwahati,Assam-781032

^3^Department of Microbiology, GMCH, Guwahati,Assam-781032

^4^Department of Biotechnology, Assam Down Town University, Guwahati,Assam-781068

Name and Address of Corresponding Author: **Dr.SubhashMedhi, Assistant Professor**

Department of Bioengineering & Technology,

GauhatiUiversity, Guwahati-781014

Email: [subhashmedhi@gauhati.ac.in](mailto:subhashmedhi@gauhati.ac.in)

Ph No: 7002485869

Name and Address of Co-Corresponding Author: **Dr. Ajanta Sharma, Professor**

Department of Microbiology,

Gauhati Medical College, Guwahati-781032

Email: [ajantasharma2002@yahoo.com](mailto:ajantasharma2002@yahoo.com)

Ph No: 9435011302

**^Ϯ^ Equal Contributing author**

**^*^Co-corresponding author**

**^**^Corresponding author**

| Table:1  **SUPPLEMENTARY DATA** | | | | | | |
| --- | --- | --- | --- | --- | --- | --- |
| Ct values obtained with Meril Covid-19 kit and In house economised RT-PCR assay | | | | | | |
| Sample serial Number | *Meril Covid-19 kit* | |  | *In house economised RT-PCR assay* | | |
|  | **Orf 1b** | **N gene** |  | **Target 1 (RdRP)** | **Target 2**  **(E gene)** | **Target 3**  **(S gene)** |
|  | 25.2 | 26.16 |  | 23.45 | 24.6 | 23.8 |
|  | 19.22 | 21.45 |  | 15.65 | 13.21 | 18.2 |
|  | 33.44 | 34.45 |  | 31.5 | 29.55 | 31.23 |
|  | 21.5 | 22.1 |  | 19.9 | 18.56 | 20.3 |
|  | 31.9 | 32.3 |  | 31.25 | 30.5 | 27.68 |
|  | 27.34 | 28.15 |  | 25.68 | 24.6 | 24.8 |
|  | 21.75 | 23.35 |  | 17.9 | 18.58 | 18.88 |
|  | 33.5 | 33.86 |  | 31.2 | 30.57 | 31.59 |
|  | 28.14 | 29.46 |  | 22.85 | 23.4 | 24.2 |
|  | 26.77 | 27.5 |  | 21.37 | 24.2 | 23.5 |
|  | 25.65 | 26.78 |  | 24 | 23.45 | 22.8 |
|  | 23.55 | 25.67 |  | 20.56 | 19.37 | 19.88 |
|  | 29.8 | 28.37 |  | 26.45 | 25.44 | 25 |
|  | 25.73 | 27.12 |  | 21.65 | 20.2 | 21.9 |
|  | 30.79 | 31.95 |  | 26.33 | 25.6 | 29.3 |
|  | 33.4 | 34.76 |  | 30.9 | 31.67 | 32.56 |
|  | 23.4 | 24.19 |  | 17.5 | 18.95 | 19.2 |
|  | 25.34 | 26.89 |  | 21.89 | 20.8 | 23 |
|  | 33.57 | 34.6 |  | 31.45 | 30.3 | 33.1 |
|  | 29.63 | 30.17 |  | 27.5 | 26.47 | 26.8 |
|  | 22.5 | 23.79 |  | 18.95 | 16.21 | 17.8 |
|  | 34.66 | 34.57 |  | 31.5 | 30.69 | 33.2 |
|  | 28.77 | 29.75 |  | 26.85 | 25.22 | 25 |
|  | 34.2 | 34.88 |  | 30.21 | 31.35 | 33.7 |
|  | 31.27 | 32.15 |  | 26.68 | 25.42 | 29.4 |
|  | 27.33 | 29.21 |  | 23.1 | 22.85 | 24.35 |
|  | 26.43 | 27.7 |  | 22.3 | 23 | 24.5 |
|  | 28.47 | 30.23 |  | 24.3 | 25.67 | 25.5 |
|  | 31.47 | 32.53 |  | 28 | 27.69 | 28.6 |
|  | 27.75 | 29.17 |  | 21.56 | 22.78 | 24.5 |
|  | 34.21 | 34.6 |  | 33.44 | 32.57 | 32 |
|  | 31.24 | 32.78 |  | 26.8 | 27.4 | 28.3 |
|  | 29.31 | 31.17 |  | 24.44 | 25.56 | 24.3 |
|  | 28.69 | 30.19 |  | 25.3 | 24.6 | 24.89 |
|  | 26.11 | 28.89 |  | 23.65 | 24.18 | 22.5 |
|  | 31.25 | 32.85 |  | 28.22 | 27.3 | 28.3 |
|  | 29.16 | 31.25 |  | 23.21 | 22.8 | 24.1 |
|  | 26.79 | 27.88 |  | 22.3 | 21.45 | 23.2 |
|  | 25.35 | 24.8 |  | 18.33 | 19.63 | 22 |
|  | 28.45 | 29.75 |  | 28.67 | 27.6 | 26.87 |
|  | 32.18 | 33.4 |  | 27.45 | 26.39 | 33.2 |
|  | 24.44 | 26.79 |  | 23.55 | 22.21 | 20.58 |
|  | 31.65 | 32.86 |  | 28.75 | 27.37 | 29.3 |
|  | 33.95 | 34.1 |  | 33.65 | 32.75 | 33.26 |
|  | 29.57 | 30.55 |  | 26.4 | 25.55 | 27.3 |
|  | 30.9 | 31.75 |  | 26.35 | 25.76 | 27.2 |
|  | 25.94 | 26.75 |  | 22.38 | 21.5 | 23.5 |
|  | 18.67 | 21.14 |  | 15.37 | 13.6 | 17.2 |
|  | 29.45 | 30.5 |  | 25.77 | 23.32 | 27 |
|  | 32.1 | 33.25 |  | 29.72 | 28.5 | 30.5 |
|  | 19.45 | 20.22 |  | 14.67 | 13.22 | 15.3 |
|  | 28.78 | 29.47 |  | 25.89 | 24.36 | 25.9 |
|  | 29.65 | 30.59 |  | 26.7 | 25.84 | 26 |
|  | 24.73 | 26.49 |  | 21.39 | 20.65 | 21.8 |
|  | 32.48 | 33.57 |  | 27.69 | 26.45 | 30.4 |
|  | 23.34 | 25.67 |  | 19.44 | 18.63 | 20.54 |
|  | 28.92 | 30.5 |  | 24.86 | 23.6 | 23.8 |
|  | 34.21 | … |  | 33.2 | 33.17 | 34.25 |
|  | 28.85 | 29.56 |  | 24.56 | 23.38 | 26.2 |
|  | 31.2 | 33.39 |  | 28.2 | 29.17 | 28.93 |
|  | 22.76 | 23.1 |  | 19.8 | 20.65 | 21.3 |
|  | 32.5 | 33.34 |  | 31.4 | 30.8 | 29.98 |
|  | 34.79 | … |  | 34.28 | 33.65 | 34.5 |
|  | 31.22 | 32.94 |  | 30.89 | 29.45 | 28.8 |
|  | 26.78 | 27.45 |  | 22.8 | 21.5 | 23.2 |
|  | 26.9 | 28.76 |  | 22.67 | 21.54 | 22 |
|  | 27.92 | 28.44 |  | 24.3 | 25.6 | 25.8 |
|  | 23.45 | 24.8 |  | 17.85 | 18.63 | 19.3 |
|  | 29.33 | 28.67 |  | 24.85 | 26.31 | 26.8 |
|  | 29.79 | 30.8 |  | 24.45 | 25.76 | 26.78 |
|  | 28.9 | 29.35 |  | 24.65 | 25.67 | 25.88 |
|  | 30.4 | 31.77 |  | 26.35 | 25.4 | 26.7 |
|  | 27.85 | 28.67 |  | 23.67 | 22.55 | 23.4 |
|  | 25.4 | 26.78 |  | 19.45 | 20.63 | 21.5 |
|  | 33.2 | 34.7 |  | 33.45 | 32.9 | 34.8 |
|  | 18.63 | 20.15 |  | 11.75 | 13.17 | 17.2 |
|  | 20.82 | 22.51 |  | 21.37 | 20.22 | 22.5 |
|  | 33.2 | 34.31 |  | 31.45 | 32.15 | 32.89 |
|  | 30.49 | 31.92 |  | 26.45 | 25.48 | 27.5 |
|  | 26.77 | 28.17 |  | 22.73 | 20.15 | 22.7 |
|  | 32.9 | 33.71 |  | 28.9 | 27.8 | 30.3 |
|  | 30.45 | 31.2 |  | 24.3 | 23.63 | 26.2 |
|  | 24.87 | 26.3 |  | 19.42 | 18.65 | 22.34 |
|  | 19.77 | 20.6 |  | 15.96 | 14.45 | 17.3 |
|  | 29.45 | 30.8 |  | 23.51 | 22.44 | 28.9 |
|  | 30.11 | 32.54 |  | 25.6 | 24.55 | 26.3 |
|  | 27.86 | 29.17 |  | 24.89 | 23.66 | 24.88 |
|  | 32.9 | 34.65 |  | 32.8 | 31.9 | 30.9 |
|  | 26.9 | 27.78 |  | 22.45 | 23.46 | 24.2 |
|  | 26.77 | 27.89 |  | 19.45 | 18.21 | 22 |
|  | 30.19 | 30.88 |  | 25.38 | 24.65 | 27.65 |
|  | 27.95 | 29.1 |  | 22.45 | 20.8 | 23.1 |
|  | 21.85 | 23.14 |  | 18.5 | 16.32 | 17.85 |
|  | 27.9 | 29.48 |  | 23.37 | 21.16 | 24.78 |
|  | 28.67 | 29.5 |  | 22.53 | 21.2 | 25.4 |
|  | 20.1 | 22.35 |  | 16.68 | 15.15 | 17.1 |
|  | 29.7 | 30.19 |  | 24.45 | 23.67 | 25.6 |
|  | 26.77 | 28.93 |  | 24.86 | 23.6 | 23.8 |
|  | 20.24 | 21.87 |  | 18.5 | 16.32 | 17.85 |
|  | 27.66 | 28.43 |  | 22.45 | 23.46 | 24.2 |

**Table 1:***Ct values obtained for N=100 SARS-CoV2 positive samples tested with Meril COVID-19 detection RT-PCR kit and our In house RT-PCR assay.*

| Dilution | Avg Ct Orf1b | Avg Ct N gene |
| --- | --- | --- |
| 10^-1^ | 23.1 | 25.62 |
| 10^-2^ | 28.75 | 30.34 |
| 10^-3^ | 33.85 | 34.32 |
| 10^-4^ | 36.02 | 38.27 |
| 10^-5^ | 39.57 | 39.95 |
| 10^-6^ | 42.5 | 43.61 |
| 10^-7^ | - | - |

**Table 2:**Average *Ct values obtained for 10-fold serially diluted SARS-CoV2 positive sample (SC005G) tested with* ***Meril COVID-19 detection RT-PCR kit****.*

**Figure 1:** *C_t_ value comparison on positive sample stratified by age Age group 80-89 showed considerably lower mean Ct value for all the three targets in comparison to overall Mean Ct which (p<0.05) is representative of high viral load.*
