# Supplementary material for: In-house reverse transcriptase polymerase chain reaction for detection of SARS-CoV-2 with increased sensitivity
Source: Sci Rep. 2021 Sep 9;11:17878. doi: 10.1038/s41598-021-97502-1 (PMC8429455; doi:10.1038/s41598-021-97502-1)
Supplement: Supplementary file 2 — Supplementary Information 2. [file 41598_2021_97502_MOESM2_ESM.pdf]

In-house Reverse transcriptase polymerase chain reaction for detection of SARS-CoV2 with increased Sensitivity.

Manash Jyoti Kalita[†,1], Kalpajit Dutta[†1], Gautam Hazarika[1], Ridip Dutta[3], Simanta Kalita[1,2], Partha Pratim Das[1,2], Manash P Sarma[4], Sofia Banu[1], Md. Ghaznavi Idris[1], Anjan Jyoti Talukdar[2], Sangitanjan Dutta[2], Ajanta Sharma[*3], Subhash Medhi[**1]

[1] Department of Bioengineering and Technology, Laboratory of Molecular Virology and Oncology, Gauhati University.Guwahati,Assam-781014
[2]Department of Medicine, GMCH, Guwahati,Assam-781032
[3]Department of Microbiology, GMCH, Guwahati,Assam-781032
[4]Department of Biotechnology, Assam Down Town University, Guwahati,Assam-781068

Name and Address of Corresponding Author: **Dr. Subhash Medhi, Assistant Professor**
Department of Bioengineering & Technology,
Gauhati Uiversity, Guwahati-781014
Email: subhashmedhi@gauhati.ac.in
Ph No: 7002485869
Name and Address of Co-Corresponding Author: **Dr. Ajanta Sharma, Professor**
Department of Microbiology,
Gauhati Medical College, Guwahati-781032
Email: ajantasharma2002@yahoo.com
Ph No: 9435011302

[†] Equal Contributing author
[*]Co-corresponding author
[**]Corresponding author

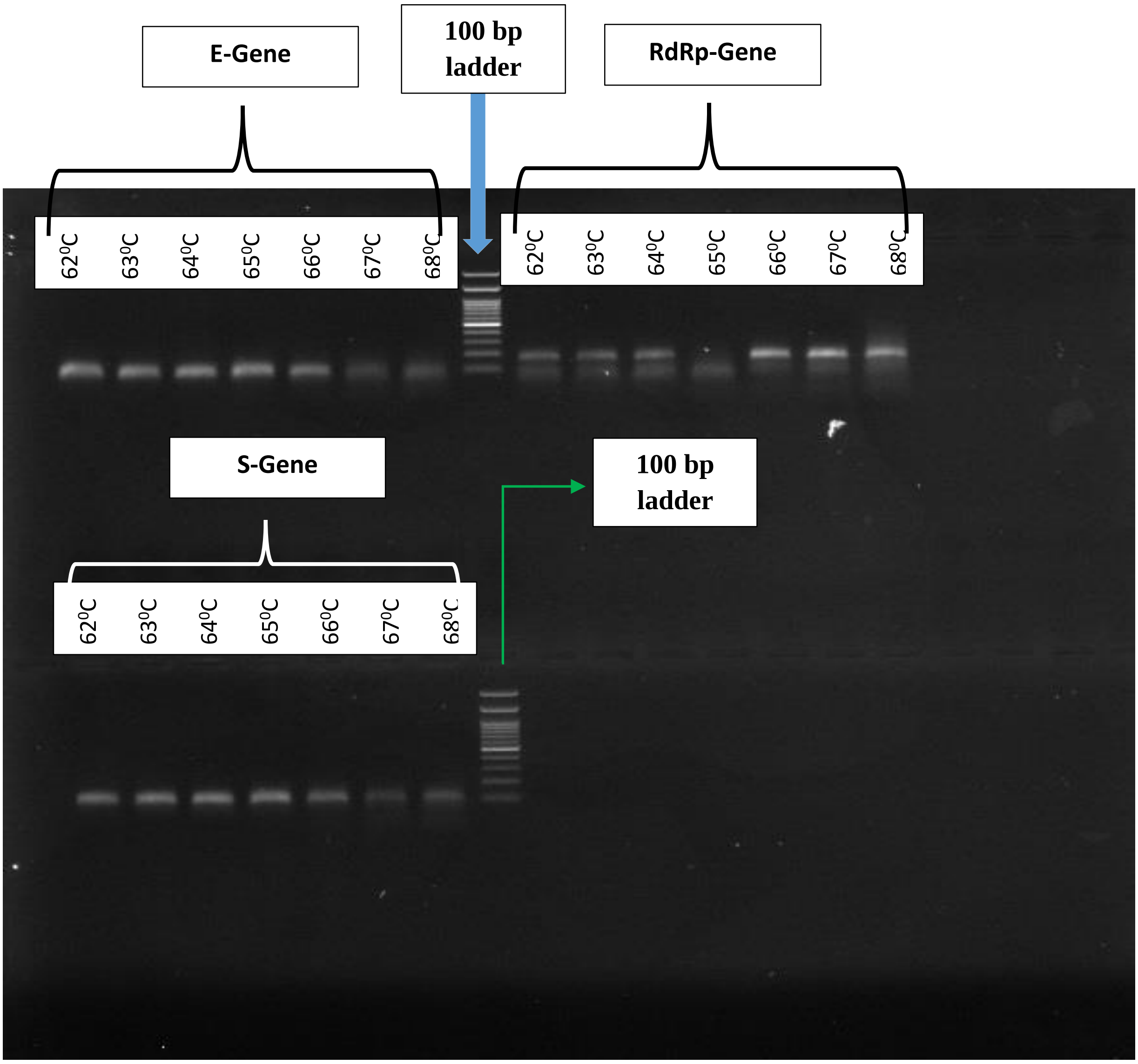

Fig 1: Amplification of E, RdRp and S genes at different temperature in gradient PCR. (Lane 1-7 E-gene, Lane 8 100bp ladder, Lane 9-15 RdRp-gene, Lane 16-22 S-gene, Lane 23 100bp ladder.
